# Dr. Yandell’s Letter

**Published:** 1867-09

**Authors:** 


### DR. YANDELL'S LETTER.

Our readers will find in the August No. of our Journal, an able and exceedingly interesting letter from London, to one of the Editors of this Journal. We will receive others, and when we do, they shall share the pleasure and instruction they afford. The writer, Dr. L. P. Yandell, Jr., is one of the ablest representatives our profession has sent abroad for many years. With a first class collegiate education, he combines the greatest thoroughness in the profession of medicine. As a sound original thinker, no man of his age deserves a higher rank; and for practical wisdom, keenness of observation, and felicity of manner, we scarcely know his equal.

The President of the Epidemiological Society of London, composed of the most learned medical men of the metropolis, complimented in very high terms a speech made by him before the society. Many of the members, also, expressed their delight at hearing him.

Dr. Yandell is the youngest son of Dr. Lunsford P. Yandell, Prof. of Chemistry in the Medical department of Transylvania University, in the glorious days of that renowned institution. He subsequently held the same chair in the Medical department of the University of Louisville. He was associated throughout his long and honorable life as a teacher with Eberle, Drake, Richardson, Short, Caldwell, Dudley, and many others. The last named and himself, are all that now remain of that bright gallaxy who were the first to exalt the standard of medical education west of the Alleghanies, and which has been felt throughout the west and south, in all the fruition of success from the lakes to the sea-board. Long may he live to enjoy a fame so justly won as a model gentleman, medical philosopher, and an earnest Christian.

